# Genomic analysis of *Elizabethkingia meningoseptica* causing bacteremia in the Brazilian Amazon

**DOI:** 10.1016/j.nmni.2024.101415

**Published:** 2024-04-16

**Authors:** Sergio Mascarenhas Morgado, Rosângela Cipriano, Fernanda dos Santos Freitas, Erica Lourenço da Fonseca, Ana Carolina Paulo Vicente

**Affiliations:** Laboratório de Genética Molecular de Microrganismos, Instituto Oswaldo Cruz, Rio de Janeiro, Brazil; São Domingos Hospital, Maranhão, Brazil; Laboratório de Genética Molecular de Microrganismos, Instituto Oswaldo Cruz, Rio de Janeiro, Brazil

Dear Editor,

*Elizabethkingia meningoseptica* (formerly *Chryseobacterium meningosepticum* and *Flavobacterium meningosepticum*) is a Gram-negative, aerobic, non-fermentative bacterium that colonizes natural and clinical environments [1]. Human *E. meningoseptica* infections are relatively uncommon, however, misidentification of *Elizabethkingia* spp. could be underestimating the real epidemiology of this species [2]. Indeed, outbreaks involving *E. meningoseptica* lineages have begun to emerge in China [1]. *E. meningoseptica* infections lead to prolonged and complicated hospitalizations due to its ability to form biofilms and intrinsic resistance to multiple antimicrobial drugs, including most β-lactams, colistin, and aminoglycosides [[1], [2], [3]]. Indeed, the *Elizabethkingia* genus encodes two chromosomal metallo-β-lactamase genes, *bla*_BlaB_ and *bla*_GOB_ (carbapenem resistance) and an extended-spectrum β-lactamase, *bla*_CME_ (cephalosporin resistance) [2,3], which negatively impact the treatment of such infections.

In a routine analysis, an *E. meningoseptica* (strain Emn14) was isolated from the blood of a young female adult (21y) in a clinical setting in the Brazilian Amazon region, showing a broad spectrum of resistance. Due to the scarcity of phenotypic and genomic data on this species, particularly in South America, we subjected this strain to whole-genome sequencing and determined its genetic relationship with other *E. meningoseptica*, also exploring its resistome and virulome.

Antimicrobial susceptibility testing of Emn14 was performed by disk-diffusion and minimum inhibitory concentration (MIC) methods and was interpreted according to the Clinical and Laboratory Standards Institute (32nd ed.). Emn14 genome was carried out using an Illumina Hiseq 2500 platform, generating reads of 150 bp. The reads were filtered (Phred score ≥20) using NGSQCtoolkit v.2.3.3 (https://github.com/mjain-lab/NGSQCToolkit), assembled with SPAdes v3.15.2 (https://github.com/ablab/spades), and analyzed with fabricate (https://github.com/tseemann/abricate) using the CARD and VFDB databases. Emn14 genome is deposited in the NCBI under the accession number JAYGGR020000000. To determine the Emn14 lineage we obtained all *E. meningoseptica* genomes from Genbank (n = 87) in December 2023 and performed a phylogenomic analysis based on their core genome using the Roary v3.13.0 (https://github.com/sanger-pathogens/Roary), snp-sites v2.5.1 (https://github.com/sanger-pathogens/snp-sites), and IQtree v1.6.12 (https://github.com/iqtree/iqtree2).

Antibiotic susceptibility testing of Emn14 showed a broad spectrum of antibiotic resistance, including amikacin, gentamicin, kanamycin, neomycin, streptomycin, tobramycin (aminoglycosides); imipenem, meropenem (carbapenems); cefalotin, cefuroxime, ceftazidime, ceftriaxone, cefotaxime (cephalosporins); spectinomycin (macrolides); ampicillin/sulbactam, amoxicillin, metilicin, penicillin, oxacillin (penicillins); norfloxacin (quinolones); sulfonamides, sulfamethoxazole (sulphonic acids); aztreonam (monobactams); tetracycline, tigecycline (tetracyclines). Susceptibility was observed only for piperacillin/tazobactam, nalidixic acid, and ciprofloxacin. For some antibiotics, the MIC was evaluated: levofloxacin (fluoroquinolone), 2ug/ml (susceptible); imipenem and meropenem (β-lactams), >32ug/ml (resistant); colistin (polymyxin), >256ug/ml (resistant); tetracycline and tigecycline (tetracyclines), 128ug/ml and 4ug/ml, respectively (resistant); chloramphenicol (phenicol), 24ug/ml (intermediate). Like other studies on *E. meningoseptica*, Emn14 was resistant to most antibiotics tested, except for some quinolones [[3], [4], [5]].

In the phylogeny, Emn14 is positioned apart from most lineages but is related to a US genome (strain CSID_3000515919; GCA_002023305) (Fig. 1; purple background). Curiously, both Emn14 and CSID_3000515919 strains infected humans in the same year (2016). The genomic comparison of these two genomes indicated that in addition to being related (Fig. 1; purple background), they have a common resistome, composed of *bla*_BlaB_, *bla*_CME_, *bla*_GOB_, *ran*A/B, *aad*S, and *cat*B genes. While CSID_3000515919 presented the *bla*_BlaB-35_ and *bla*_GOB-44_ alleles, Emn14 had alleles most similar to *bla*_BlaB-19_ (99.47 % identity) and *bla*_GOB-27_ (99.43 % identity). Considering their virulome, both shared three genes, *cap*8E (immune modulation), *gro*EL (adherence), and *tuf*A (adherence), however, Emn14 also had two more genes, *cap*8G and *neu*B genes (both related to immune modulation). Except for the *neu*B gene, Emn14 presented a common virulome with Chinese/French genomes (*gro*EL, *tuf*A, *cap*8E, *cap*8G) that belong to a lineage that persisted from 1982 to 2018 (Fig. 1; green background).

**Fig. 1:**
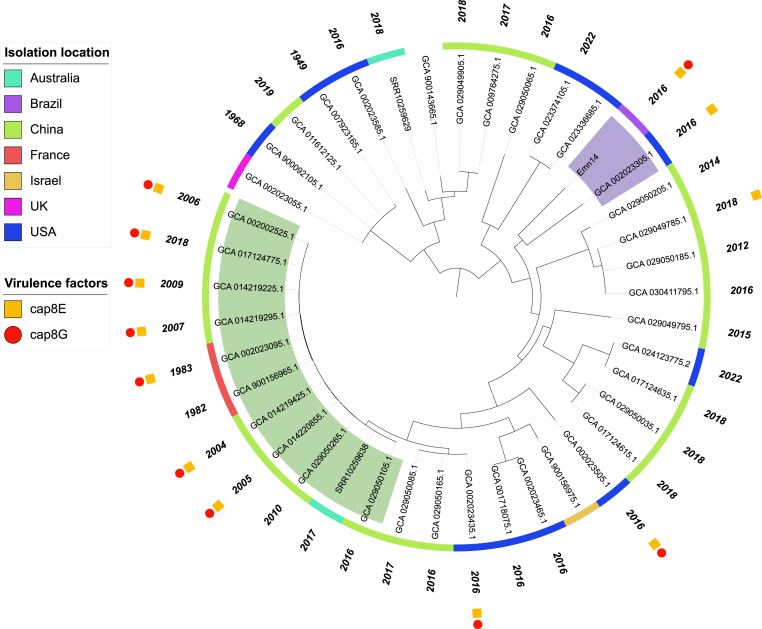
Maximum-likelihood tree of 41 *E. meningoseptica* genomes. This tree was generated with fewer genomes than the initial 87 to simplify it. Genomes representing all observed lineages were maintained. The best evolutionary model was TVM + F + ASC + R2, selected by the Bayesian information criterion. The coloured blocks indicate the location of isolation of the genomes. The purple and green background clusters highlight the Emn14 and CSID_3000515919 clade and the persistent *E. meningoseptica* lineage, respectively. Red circles on branches represent >70 % bootstrap.

Here we provided an epidemiological scenario in which a Brazilian *E. meningoseptica* strain is related to a genome from the USA. Indeed, based on evolutionary analysis, the USA may be the most likely source of pathogenic *E. meningoseptica* [1]. In this way, our study provides evidence about the dispersion of *E. meningoseptica* strains also between the Americas. Furthermore, surprisingly, the Emn14 strain was isolated from the blood of a young adult, which is even rarer considering that most infections caused by this species are associated with neonates and immunocompromised patients [2]. All these data highlight the need for genomic analyses to survey and understand the current new epidemiological scenario involving *E. meningoseptica*.

## Funding statement

This study was financed by FAPERJ - Fundação Carlos Chagas Filho de Amparo à Pesquisa do Estado do Rio de Janeiro, Processo SEI-260003/019688/2022.

## CRediT authorship contribution statement

**Sergio Mascarenhas Morgado:** Formal analysis, Methodology, Writing – original draft, Writing – review & editing. **Rosângela Cipriano:** Investigation, Resources. **Fernanda dos Santos Freitas:** Investigation. **Erica Lourenço da Fonseca:** Writing – review & editing. **Ana Carolina Paulo Vicente:** Conceptualization, Funding acquisition, Methodology, Writing – original draft, Writing – review & editing.

## Declaration of competing interest

The authors declare that they have no known competing financial interests or personal relationships that could have appeared to influence the work reported in this paper.
